# The contrasting hidden consequences of parasitism: Effects of a hematophagous nematode (*Uncinaria* sp.) in the development of a marine mammal swimming behavior

**DOI:** 10.1002/ece3.4914

**Published:** 2019-03-07

**Authors:** Felipe Montalva, Diego Pérez‐Venegas, Josefina Gutiérrez, Mauricio Seguel

**Affiliations:** ^1^ Facultad de Ciencias Biológicas Pontificia Universidad Católica de Chile Santiago Chile; ^2^ PhD Program in Conservation Medicine, Facultad de Ciencias de la Vida Universidad Andrés Bello Santiago Chile; ^3^ Instituto de Patología Animal, Facultad de Ciencias Veterinarias Universidad Austral de Chile Valdivia Chile; ^4^ Programa de Investigación Aplicada en Fauna Silvestre, Facultad de Ciencias Veterinarias Universidad Austral de Chile Valdivia Chile; ^5^ Department of Pathology, College of Veterinary Medicine University of Georgia Athens Georgia; ^6^ Present address: Odum School of Ecology University of Georgia Athens Georgia

**Keywords:** *Arctocephalus australis*, hookworms, marine mammal, parasitism, swimming behavior, *Uncinaria* sp

## Abstract

1. Parasites are an important part of ecosystems, playing a critical role in their equilibrium. However, the consequences of parasitism beyond the direct effects associated with disease and mortality are not completely understood. This gap in knowledge is in part due to the difficulties to isolate the effect of single parasite species on physiological and behavioral traits in natural systems.
2. The South American fur seal (*Arctocephalus australis*)–hookworm (*Uncinaria sp*.) interaction offers an ideal system to overcome these difficulties and study the behavioral and physiological effects of parasites in their hosts.
3. Hookworms cause stunted growth and anemia in pinniped pups, which could affect early life active behaviors such as swimming. The aim of this study was to determine the effects of hookworms (*Uncinaria* *sp*.) on the development of swimming capabilities in *A. australis* through physiological and ethological analyses.
4. Higher parasite burden was associated with reduced growth rates and lower blood hemoglobin concentrations, whereas scaled body mass and blood hemoglobin levels had an important positive effect on the water activity of the pups. However, antihookworm treatment did not affect the level of water activity of the pups, and pups with high hookworm burden increased their time budget in water. This was probably related to lower maternal attendance in heavily parasitized pups, leaving these pups more time to perform water activities. Therefore, pups with heavy hookworm burden, despite having decreased growth rates and blood hemoglobin concentrations, compensated for their handicap in physiological traits related to swimming by spending more time in the water.
5. This work offers new insights to understand the contrasting effects of parasites on aquatic organisms, and the compensatory mechanisms employed by infected animals to avoid the worst consequences of parasitism.

## 1 INTRODUCTION

Parasites are organisms that extract resources from a host in order to reproduce (Schmid Hempel, 2011). Therefore, all true parasites have a negative impact for the host because they use resources that would be otherwise available for the parasitized organism. However, the effect of parasites at the population and ecosystem level can be more complex. Parasites are important components in ecosystems, playing key functions in their balance, for instance altering energy flows or selecting individuals, having a variable (positive or negative) long‐term effect on populations and ecosystems (Hudson, Dobson, & Lafferty, 2006). However, detecting these dynamics in natural systems is challenging because the impact of parasites on host populations is sometimes subtle and difficult to document (Pedersen & Fenton, 2007). These subtle or “hidden effects” of parasitism are hard to measure because they are associated with changes in behavior and physiological variables which may be affected by factors other than parasitism (e.g., diet, maternal care, genetics) (Carlsson et al., 2017; Higgins & Gass, 1993; Seguel & Gottdenker, 2017). Additionally, the changes in an animal physiology and health status due to parasitism are related not only to the parasite but also to the host response to the infection (Ezenwa et al., 2016; Fox, 1997; Seguel et al., 2017). These responses include physical, behavioral, and functional changes that can be quite variable between individuals and populations. This heterogeneity can result in contrasting effects of parasites on animal populations, and it is one of the foundations for the adaptive models on the evolution of parasitism (Schmid Hempel, 2011; Vanderwaal & Ezenwa, 2016). However, it is not well understood how and in which context adaptive responses to parasitism are developed by the host.

Aquatic air‐breathing vertebrates need to develop swimming and diving abilities to obtain their food items in their environment (Castellini & Mellish, 2015). The development of these skills results from interactions between animal behavior and physiology, which change with age, body size, reproductive status, and other life‐history traits (Gastebois, Viviant, & Guinet, 2011; Lea, Johnson, Melin, Ream, & Gelatt, 2010; Verrier et al., 2011; Weise & Costa, 2007). Among marine mammals, pinnipeds mix foraging in ocean with reproduction on shore, and they may exhibit different strategies and adaptations to guarantee their survival in these environments (Franco‐Trecu, García‐Olazábal, Tassino, & Acevedo, 2016; Verrier et al., 2011). In this context, otariids (fur seals and sea lions) and odobenids (walruses) display long periods of maternal dependency (income breeders) when pups must learn to swim and forage before weaning (Schulz & Bowen, 2004), while phocids (true seals) show strategies that allow pups to swim soon after birth (capital breeders) (Carter et al., 2017). Therefore, the development of physiological conditions for foraging (i.e., swimming skills) plays a key role in offspring nutritional independence and survival during the transition from land to sea (Baylis et al., 2005; Somo, Ensminger, Sharick, Kanatous, & Crocker, 2015).

Most of the studies addressing mammalian swimming ontogeny focus on the evolution of swimming skills through time, and how they are influenced by variables such as maternal care strategies (Fowler, Costa, Arnould, Gales, & Kuhn, 2006) and individuals’ growth rates (Guinet, Servera, Deville, & Beauplet, 2005). However, little is known regarding the impact of other external factors such as parasitism on the swimming ontogeny of mammals.

On Guafo Island, Northern Chilean Patagonia inhabits the South American fur seal (SAFS, *Arctocephalus australis*), an otariid species. Pups of this species experience mortality due to several causes, but hookworm disease, caused by the hematophagous parasite *Uncinaria *sp., is the main cause of death among SAFS pups on Guafo Island (Seguel et al., 2017; Seguel, Pavés, Paredes, & Schlatter, 2013). *Uncinaria sp*. (Ancylostomatidae) is delivered from the mother to pups through the milk, stays in the small intestine of the pups for 2 months, and then the parasite either dies along with its host or is cleared from the gut through an immune‐mediated mechanism (Seguel & Gottdenker, 2017; Seguel, Montalva et al., 2018; Seguel et al., 2013). In many otariid populations, the prevalence of hookworm infection is close to 100% (Seguel & Gottdenker, 2017), and total mortality can be up to 20% of all pups born in a season (Lyons et al., 2011; Seguel, Montalva et al., 2018). This high mortality is associated with the aggressive feeding behavior of the adult parasites, which cause deep wounds in the host intestine to feed on blood (Seguel et al., 2017; Seguel, Muñoz et al., 2018). Therefore, besides mortality, significant effects on health and physiological parameters such as anemia and stunted growth are observed with this parasite (Marcus, Higgins, & Gray, 2015; Seguel, Muñoz et al., 2018). Additionally, since this parasite affects neonates, it could impair the development of physiological and behavioral processes in otariids. For instance, in New Zealand sea lion pups (*Phocarctos hookeri*), animals treated with ivermectin (an antiparasitic drug) exhibit better growth rates and increased long‐term (years) survival than animals infected with hookworms when they were pups (Chilvers, Duignan, Robertson, Castinel, & Wilkinson, 2009; Michael, Chilvers, Roe, & Gartrell, 2015). These findings suggest that this parasite affects long‐term recruitment of individuals in the population despite causing an acute, short‐term infection. However, it is unknown how hookworms could affect long‐term survival in pinnipeds, after the acute infection phase has been resolved.

Pinnipeds depend on enhanced aerobic diving capacity to support foraging behaviors (Costa, Kuhn, Weise, Shaffer, & Arnould, 2004); therefore, significant diving activity starts when pups have reached a minimum body size and standard levels of hemoglobin, which are essential for oxygen transport and aerobic capacity (Horning & Trillmich, 1997; Somo et al., 2015). In Antarctic fur seal pups (*Arctocephalus gazella*), development of diving skills is strongly influenced by body length and its relation to blood hemoglobin levels (McCafferty, Boyd, & Taylor, 1998), and in Northern fur seal pups (*Callorhinus ursinus*), body size and body condition influence their diving capability prior to weaning (Lea et al., 2010). Therefore, physiology traits and size could be significant in the ontogeny of swimming behavior in otariids neonates, and given the known effects of *Uncinaria *sp. on individual physiology (e.g., anemia, retarded growth), it is possible to predict that parasitism by *Uncinaria* sp., an hematophagous nematode, could be associated with a decrease in swimming activities by affecting the pups growth/body size and hemoglobin levels. Additionally, hookworm disease affects SAFS pups when they are between 30 and 60 days old (Seguel, Muñoz et al., 2018; Seguel et al., 2013), which is the time when other otariid species start to develop water behavior (Horning & Trillmich, 1997; McCafferty et al., 1998).

Given the mentioned particularities in the fur seal hookworm transmission dynamics, and the possibility to completely cure infection with a single anthelmintic dose in newborn pups (Seguel, Muñoz et al., 2018), the SAFS–hookworm system is ideal to understand the behavioral and physiological effects of parasitism in natural settings. This study aimed to describe the ontogeny of swimming in a mammal in the context of parasitism. Specifically, we aimed to determine (a) the nature of swimming ontogeny in SAFS pups and (b) whether *Uncinaria* sp. affects early development of swimming behavior in SAFS pups.

## 2 MATERIALS AND METHODS

### 2.1 Study site and animal handling

The study was performed in the breeding colony of SAFS at Guafo Island, Northern Chilean Patagonia (43°35′34.9′′S, 74°42′48.53′′W), between 14 December 2016 and 4 March 2017. Pups were captured by hand, three to seven times (mean = 5.2 ± 2.3 captures per pup), during the study period (12 weeks). At the first capture, pups were marked in the back with a number using commercial hair decoloring solution. Additionally, when they were at least 1 month old, they were tagged in the pectoral flipper with a plastic tag (Allflex^®^; Allflex Inc., TX, USA). In each capture session, standard length, weight, and sex were recorded, and a fecal swab was obtained and saved in tubes with Sheather's sucrose solution for later hookworm (*Uncinaria* sp.) egg counting in the field laboratory (Seguel, Muñoz et al., 2018). Additionally, in order to quantify the effect of parasites on pups’ physiological traits, a random group of pups were treated with a subcutaneous dose of the antiparasitic drug ivermectin (300 µg/kg) when pups were between 1 and 7 days old (control group). Hookworm prevalence is approximately 100% among fur seal pups at Guafo Island and hookworms begin developing in the intestine of 1‐week‐old pups (Seguel, Muñoz et al., 2018). Additionally, since treated pups did not become re‐infected throughout the study (based on the repeated cropological and clinical examinations), we considered these treated pups as a good control (hookworm‐free) group (Seguel, Muñoz et al., 2018). In every capture, blood was collected from the caudal gluteal vein of pups into EDTA Vacutainer® tubes in order to determine pups’ hemoglobin concentrations in the field laboratory using previously described methods for this animal population (Seguel et al., 2016). Growth rates were obtained by dividing the weight difference between the first and last capture by the total days between those captures.

The minimal number of days used to obtain growth rates was estimated in a period of 20 days because a linear function can be assumed (Doidge, Croxall, & Ricketts, 1984). Pup scaled body mass index (SMI) was determined by the division between the values of weight and length (Peig & Green, 2010). The pups that did not complete the whole study period (e.g., mortality) were excluded of the analysis.

### 2.2 Swimming behavior

Daily observations of marked pups were conducted from elevated points in the rookery for 1.5 hr in the morning and 1.5 hr in the afternoon. Pup behavior was identified by focal sampling (Altmann, 1974) and recorded in ethograms. Pup activities were categorized into activities on land such as receiving maternal care (pups in the presence of their mothers, suckling or resting), spots (groups bigger than three pups engaged in activities such playing), and resting (pup alone or in group resting without presence of its mother in the rookery) and activities in water according to previously stablished criteria (resting, exploring, swimming, playing, Table 1). Similarly, focal sampling was used to measure the diving time of a few (*n* = 12) pups by recording how long the animal remained submerged in a total period of 10 min. For these observations, diving was recorded when at least 80% of the animal's body, including the head, was submerged under water. The inland social interaction (i.e., pup spots occurrences) between pups was observed and recorded to determine whether the frequency of the interactions was related to the pup's parasitic burden. In order to determine the time that pups spend in water, we calculated the proportion between events in water and events in land in different groups of pups divided according to their parasitic burden. Pups that died, without ethological data and/or non‐observed for a period of more than 1 week, were excluded from the analysis.

**Table 1:** Description of activities preformed on water by *Arctocephalus australis* pups

Behavior	Description
Resting (Re)	The animal remains inactive (e.g., sleeping) with the body in contact with the water of the intertidal pools
Exploring (Ex)	The animal wanders in the intertidal pools stopping to submerge the head briefly
Playing (Pl)	The animal is in intertidal pools in company of others pups engaging in group games (e.g., fighting like adults)
Swimming (Sw)	The animal is totally submerged in the intertidal pools moving with the flippers to displace fast across the water

### 2.3 Data analyses

To determine the effect of *Uncinaria* sp. on pup growth rates, SBM, and hemoglobin levels, the animals were separated into groups according to their parasitic burden as determined by a semiquantitative fecal eggs count protocol developed and standardized for the studied fur seal rookery (Seguel, Muñoz et al., 2018). Pups with more than five hookworm eggs per slide, in any capture, were categorized as “severe infection,” whereas animals with less than five eggs per slide were categorized as mild infection. Previous studies have shown that this cutoff value represents intestinal burdens (120–250 nematodes) related with noticeable changes in the small intestine (Seguel et al., 2017; Seguel, Muñoz et al., 2018). Additionally, pups treated with ivermectin were handled as a control (hookworm‐free) group. To test the differences in SBM and hemoglobin between groups, one‐way ANOVAs were applied with a post hoc Tukey's test. For these comparisons, we selected the measurement from each pup obtained when pups show less variability in these measurements because of age (seguel et al., 2016), and when they start to show active water behavior (February). Correlations between hookworm (burden, infection group) and host‐related (SMI, growth rate, hemoglobin, maternal care received, water activity, frequency of “spots”) variables were tested according to data distribution. The behavioral records were sorted into tables and classified according to their frequencies of occurrence. To determine differences in the behavior frequencies of occurrence and growth rates between sexes, generalized linear models (GLMs) with Poisson distribution were fitted.

In order to determine the effect of *Uncinaria* sp. on the development of aquatic behavior of SAFS pups, we classified the swimming events as present (1) if they were recorded at least once in a pup and as absent (0) if this behavior was not recorded in pups during the study period. Therefore, pups active water behavior (swimming) was handled as a binomial variable. Binomial GLMs were fitted with the pup swimming activity as the response variable and pup parasitic burden, hemoglobin levels, growth rates and scaled body mass as the predictors in the full model. Different models were fitted, and final models were ranked and selected based on second‐order Akaike information criteria (AICc). Predictors effect was considered significant at the *p* < 0.05 level.

To determine the effect of hookworms in the overall water activity of pups, the proportion of time spent by pups in water and land was calculated by dividing the total number of events in water or land by the total number of events (in land and water) recorded for that pup. The level of maternal care received was excluded because previous studies have shown that nursing events modify the fur seal pups hookworm burden through modulation of the hookworm immune clearance (Seguel, Montalva et al., 2018). Differences in the proportion of time spent in water between pups with different hookworm burdens (mild vs. severe) and controls were tested by fitting a GLM with the proportion of time in water as response and group as a categorical predictor (mild, severe, and control). In order to determine whether the proportion of pups showing active water behavior (playing and/or swimming) was related to their hookworm infection status, the differences in the presence or absence of active water behavior between groups were assessed through binomial GLMs. A similar approach was used to test the proportion of pups showing playing or swimming behavior between different groups. If differences between pups with mild and severe infection were not significant and treated pups showed a difference with these two groups, pups were separated in “infected” and “treated” (noninfected), and the differences between these groups were tested through a binomial GLM using hookworm infection status as a categorical predictor.

All statistical analyses were performed using “R version 3.3.3” statistical software (R Core Team 2017).

## 3 RESULTS

### 3.1 Ontogeny of swimming in South American fur seal pups

**Figure 1:**
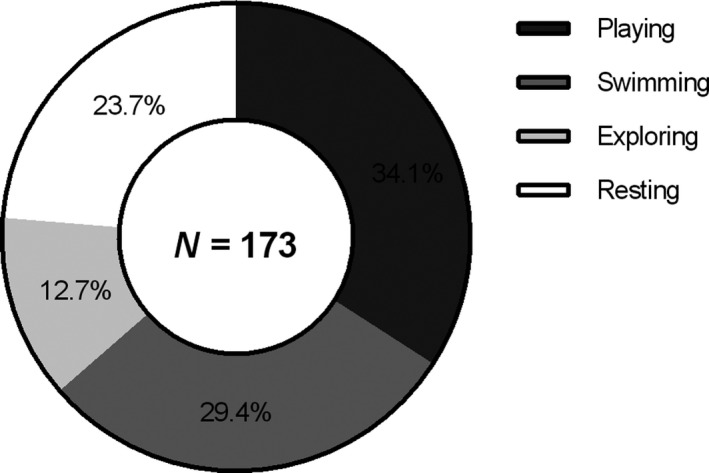
Total of recorded water events for 56 South American fur seal (*Arctocephalus australis*) pups. Resting (Re), exploring (Ex), playing (Pl), and swimming (Sw) events

For a period of 86 days, aquatic behaviors were observed in 56 pups (24 females and 32 males) with a total of 173 events recorded. Of these observations, 59 (34.1%) corresponded to playing events, 51 (29.5%) to swimming events, 41 (23.7%) to resting events, and 22 (12.7%) to exploring events (Figure 1). There were no significant differences between the number of events recorded for females and males (GLM, 2.1 ± 0.24, *t* = 6.4, *p* = 0.78).

**Figure 2:**
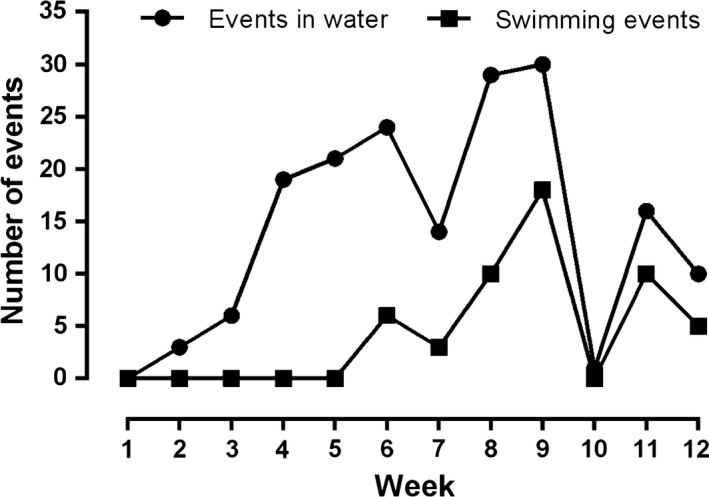
Frequency of total number of events in water, and swimming events for *Arctocephalus australis* pups across 12 weeks between December and March of the 2017 Austral summer in Guafo Island, Northern Chilean Patagonia

The first record of pups in water (playing, resting) occurred the second week of study, 1 week after the pupping peak (15 December, Pavés & Schlatter, 2008), and the first record of swimming occurred at the sixth week of study. These behaviors always occurred at the intertidal pools (Supporting information Video S1), and incursions of pups in open waters were never observed during the duration of the study. The peak of events in water (playing, swimming, resting) were registered at the ninth week of observations (8–15 February, *n* = 29) (Figure 1). However, the following week (10th), there were no records of water activity, probably related to a longstanding storm that eliminated intertidal pools and decreased visibility (Figure 2). Pups were observed 66.2% of the time in spots, and there was a positive correlation between the frequency of this social interaction and number of playing events in land (Spearman rho, *r* = 0.71, *p* < 0.001) and the number of swimming events (Spearman rho = 0.62, *p* = 0.001).

Pups started to show diving behavior during the fifth week of study (late January), when pups are on average 5 weeks old. Dive times were obtained for 11 pups with a mean of 4.01 ± 1.9 min, a maximum of 8.4 min, and a minimum of 1.5 min of immersion within a period of 10 min (Supporting information Table S1). The dive times were not correlated with the scaled body mass (SBM) of pups (12.1 ± 1.4, *n* = 9) (Pearson's correlation, *r* = −0.38; *p* = 0.31) or with hemoglobin levels (10.4 ± 2.1 g/dl) (Pearson's correlation, *r* = −0.19, *p* = 0.63). A positive correlation existed between pups lengths (77 ± 2.6 cm) and hemoglobin levels (Pearson's correlation, *r* = 0.78, *p* = 0.013).

Individual fur seal pups differed in the type of water activity showed throughout the study. Forty‐seven (84%) pups were observed playing and/or swimming (active behavior) at least once during the study. Nine (16%) pups were observed only resting and/or exploring in water (passive behavior). Specifically, in relation to active water behavior, 28 (50%) pups were observed swimming at least once during the study period.

### 3.2 Effect of hookworms (*Uncinaria* sp.) on physiological traits of pups

**Figure 3:**
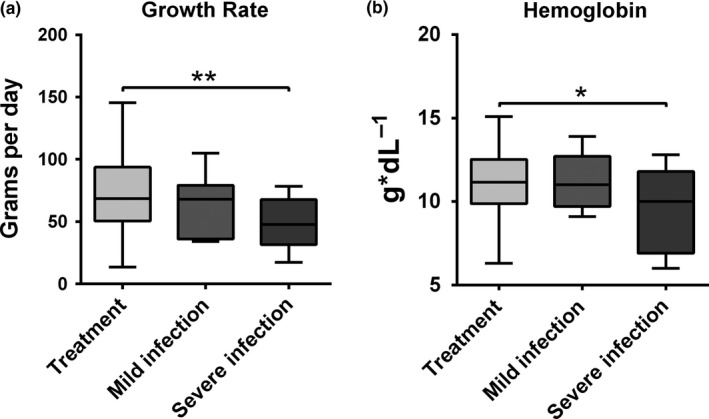
Growth rates (a) and hemoglobin levels (b) for different groups of South American fur seal (*Arctocpehalus australis*) pups. Pups with severe infection had lower growth rates than pups treated with ivermectin (controls) (one‐way ANOVA, *df* = 2, *F* = 5.12, *p* = 0.008, Tukey multiple comparison test, *p* = 0.005). Similarly, pups with severe infection had lower hemoglobin concentration than controls (one‐way ANOVA, *df* = 2, *F* = 3.89, *p* = 0.024, Tukey multiple comparison test, *p* = 0.018). **p = 0.001–0.01, *p = 0.01–0.05

Grow rates were measured in 94 pups (41 females and 53 males), with no differences between sex (GLM, 1.1 ± 0.12, *t* = 4.4, *p* = 0.72). There was a negative correlation between growth rate and pup parasitic burden (Spearman rho, *r* ^2 ^= −0.25, *p* = 0.014). Significant differences in growth rates existed between ivermectin‐treated pups (68.3 ± 32.3 g/day, *n* = 34) and pups with severe infection (47.1 ± 26.1 g/day, *n* = 37) (one‐way ANOVA, *df* = 2, *F* = 5.12, *p* = 0.008, Tukey multiple comparison test, mean = 780.1, rank = 3.4, *df* = 91, *p* = 0.005) (Figure 3a). SMI was similar in pups with different hookworm infection status (GLM, severe infection = 0.13 ± 0.52, *t* = 0.26, *p* = 0.79; treated pups = 0.42 ± 0.46, *t* = 0.92, *p* = 0.36). A significant negative correlation existed between hemoglobin levels and pups parasitic burden (Spearman rho, *r* = −0.3, *p* = 0.007). There were significant differences between hemoglobin levels of ivermectin‐treated pups (11.7 ± 1.9 g/dl, *n* = 34) and pups with severe infection (10.7 ± 2.1 g/dl, *n* = 37) (one‐way ANOVA, *df *= 2, *F* = 3.89, *p* = 0.024, Tukey multiple comparison test, mean = 4.1, rank = 3.4, *df* = 91, *p* = 0.018) (Figure 3b).

### 3.3 Effect of hookworms (*Uncinaria* sp.) on the social and water behavior of fur seal pups

The number of social events in land (spots and playing) decreased with higher hookworm burdens (GLM, −0.43 ± 0.11, *Z* = −3.8, *p* = 0.0001). The presence or absence of swimming behavior in the pups was positively conditioned by hemoglobin levels and SMI (GLM, 23.1 ± 10.3, *Z* = 2.23, *p* = 0.03); however, the interaction between these two predictors was important as well (Table 2). The effect of parasite burden over pup active aquatic behavior was not significant (GLM, −0.08 ± 0.07, *Z* = −1.05, *p* = 0.3); however, this variable was retained in the top‐ranked model (Tables 2 and 3). Pup growth rate was not included in the top two models (Table 3).

**Table 2:** Log likelihood (LogLik) and Akaike information criteria corrected (AICc) for selected GLM with binomial distribution with the presence or absence of swimming activity as response. Hemoglobin levels (Hg), scaled body mass (SBM), growth rates (GR), and hookworm burden (HB) are the predictors

Model	Predictors	AICc	Delta AICc	LogLik	*df*	*p*‐value
1	Hg + HB + SBM + Hg:SBM + Hg:HB	99.5	0	−83.2	7	0.01
2	Hg + HB + SBM + Hg:SBM +HB:SBM	101.3	1.7	−84.9	7	0.01
3	Hg + GR + HB + SBM + Hg:SBM + HB:SBM	103.8	4.3	−84.8	8	0.02
4	Hg + GR + HB + SBM + Hg:HB + Hg:SBM + HB:SBM	104.4	4.9	−82.5	9	0.03
5	Hg + GR + HB + SBM + Hg:HB + GR:Eggs + Hg:SBM + HB:SBM	107.2	7.7	−82.4	10	0.03

**Table 3:** Description of the best‐fit binomial GLM model with presence or absence of swimming activity in *Arctocephalus australis* pups as response and the coefficients of each term

Term	Estimate	Standard error	*Z*	*p*
Intercept	23.1	10.3	2.22	0.01
HB	−0.1	0.1	−1.04	0.3
SBM	−2.1	0.9	−2.34	0.01
Hg	−1.7	0.8	−2.01	0.04
Hg:HB	0.01	0.01	1.34	0.2
Hg:SBM	0.2	0.1	2.14	0.03

Note

HB: hookworm burden, Hg: hemoglobin levels; SBM: scaled body mass.

**Figure 4:**
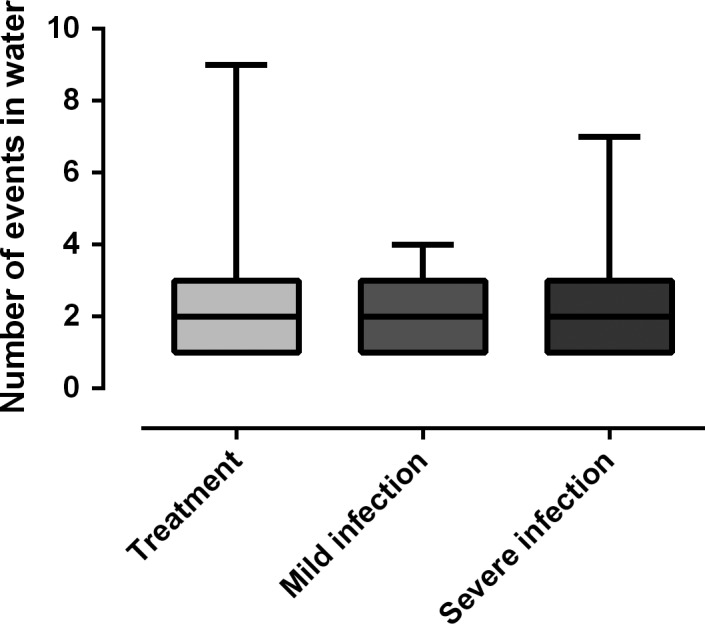
Number of events in water according to the hookworm infection status of South American fur seal (*Arctocephalus australis*) pups. There were no differences between groups of pups (GLM, 0.6 ± 0.2, *Z* = 2.9, *p* = 0.3)

**Figure 5:**
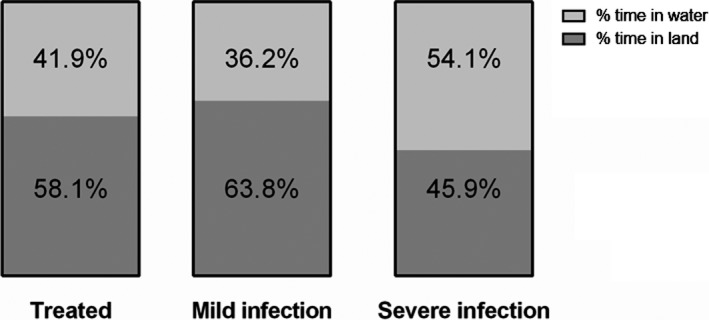
Time budget of *Arctocephalus australis* pups according to their hookworm infection status. Pups with severe hookworm infection spend more time in water (GLM, 0.8 ± 0.3, *t* = 2.66, *p* = 0.01)

**Figure 6:**
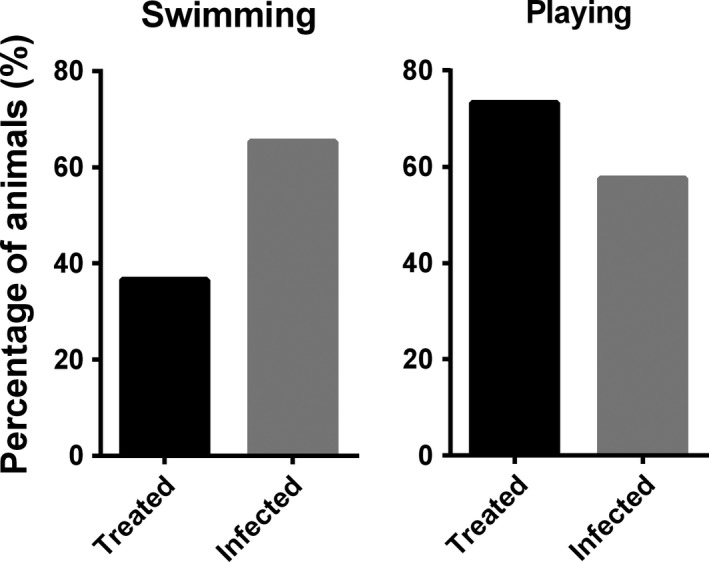
Proportion of hookworm‐infected and hookworm‐free (treated) South American fur seal (*Arctocephalus australis*) pups that showed swimming and playing behavior in water

There were no differences in the total number of events in water between pups with severe and mild hookworm infection and controls (GLM, 0.6 ± 0.2, *Z* = 2.9, *p* = 0.3; Figure 4). However, pups with severe infection spend 54.1% of their time in water, whereas pups with mild infection and treated spend 36.2% and 41.9% of their time in water, respectively (GLM, 0.8 ± 0.3, *t* = 2.66, *p* = 0.01; Figure 5). Pups with severe infection also registered a lower number of maternal care events, compared to controls and pups with mild infection (GLM, −0.8 ± 0.6, *t* = −1.42, *p* = 0.02). In the multimodel analysis, the pups with higher scaled body mass (GLM, 0.12 ± 0.05, *Z* = 2.3, *p* = 0.021) and fewer maternal care events (GLM, −1.9 ± 0.89, *Z* = −2.12, *p* = 0.034) tended to have more behavior events recorded in water (Table 4). Approximately 65% of the hookworm‐infected pups registered at least one event of swimming, whereas 35% of hookworm‐free pups (treated with ivermectin) registered at least one swimming event (GLM, infected pups = 1.18 ± 0.55, *Z* = 2.11, *p* = 0.035). The opposite occurred with respect to playing events in water with a higher proportion of hookworm‐free pups (73.3%) displaying this behavior compared to infected pups (57.6%) (Figure 6); however, the differences between infected and noninfected pups were not significant (GLM, infected pups = −0.70 ± 0.57, *Z *= −1.22, *p* = 0.22).

**Table 4:** Coefficients and significance level of predictors for selected models (GLM with Poisson distribution) with total number of events in water as response. Models are ranked based on second‐order Akaike's information criteria (AICc)

Model	Intercept	Predictors								*df*	logLik	AICc	Delta AICc	Weight
		SBM	GR	HG	HW Burden	Nursing	Treatment	BCI:HG	GR:HG					
1	−0.231**	0.1181*		0.0522		−1.91*				4	−121.8	252.4	0	0.3
2	0.148*	0.1303*				−1.72				3	−123.2	252.9	0.487	0.24
3	−0.080*	0.1050*		0.0479		−1.94*	0.133			5	−121.4	254	1.532	0.14
4	1.519*	−0.0245*		−0.1058		−1.84*		0.013		5	−121.7	254.5	2.102	0.11
5	0.973*				0.0007	−1.89*	0.167	0.005		5	−121.7	254.6	2.159	0.1
6	−0.075*	0.1052*		0.0475	−0.0001	−1.94*	0.131			6	−121.4	256.5	4.045	0.04
7	1.680*					−1.42*				2	−126.4	257.1	4.647	0.03
8	0.198*		0.0147	0.1283		−1.96*			−0.001	5	−123.2	257.6	5.204	0.02

Note

GR: growth rate; HW burden: hookworm burden; nursing: number of nursing events; SBM: scaled body mass; treatment: ivermectin (antiparasitic) treatment.

*p = 0.01–0.05 **p < 0.01

## 4 DISCUSSION

### 4.1 Ontogeny of swimming of SAFS pups

With regard to the number of events that occurred in water, playing and swimming predominated, which are mainly linked to the development of pups’ social capabilities in the early stage of their life (Gastebois et al., 2011). Thus, under natural conditions, the pups first learn to play in puddles while developing more skilled water behaviors, which suggest the allocation of the social activity time budget of pups while mothers are absent (Chechina, Kovalenko, Kulagina, & Mikhailenko, 2004). With regard to swimming events, this behavior began to manifest at the sixth week of observations, when pups are on average 1 month old according to Guafo Island pupping peak (Pavés & Schlatter, 2008). This is similar to what has been reported in other otariid species, such as Antarctic fur seal pups, which start to swim in shallow pools at the first month of life (McCafferty et al., 1998). In this study, fur seal pups were regularly observed swimming together in large groups, and therefore, it is likely that social learning is a major part of early activity at sea. The decrease in the number of events in water at the seventh week of observation coincides with the acute phase of *Uncinaria* sp. infection in fur seal pups (Seguel, Muñoz et al., 2018; Seguel et al., 2013). During this period, the most detrimental effects of hookworms on fur seal pups such as tissue damage, peritonitis, and anemia are observed (Seguel et al., 2017, 2011); therefore, it is possible that pups at this period reduce more active behaviors while coping with the worst consequences of hookworm infection instead of allocating energy to swimming activities. The reduced number of land social events in fur seal pups with severe hookworm infection supports this hypothesis and suggests that *Uncinaria sp*. could have an effect on the social behavior of fur seal pups.

The lack of behavioral events in water during the 10th week of study was related to a longstanding storm, which eliminated intertidal pools and decreased visibility. In similar studies, it has been shown that extreme weather can have significant effects on water behavior and even cause mortality of animals that despite these phenomena engage in water activity (Baker & Donohue, 2000; Seguel et al., 2011). The lower number of water events in the following 2 weeks could be related to molting, which usually starts around this time in fur seals at Guafo Island (Paves & Schlatter 2008). In Antarctic fur seals, a decrease of up to 35% in water activity has been recorded because of molting (Baker & Donohue, 2000).

Exploring activities were observed in few cases and never in spots or with the mother onshore, which is probably attributed to the fact that these individuals appeared to be looking for a place to rest or calling their mothers. Resting was always evident after the pup was fed by the mother, and this type of conduct is considered an energy saving and thermoregulation strategy (Guinet et al., 2005).

### 4.2 Effect of hookworms (*Uncinaria* sp.) on physiological traits and swimming behavior of pups

Pup growth rates were influenced by hookworm burden, and pups with severe infection showed slower growth rates than ivermectin‐treated pups. This negative impact of *Uncinaria* sp. on the growth of pups could be explained by the efficiency of the parasite to extract resources from the host for its own growth and reproduction (Fox, 1997; Seguel et al., 2017; Seguel, Muñoz et al., 2018). In this context, our findings are similar to other studies that have measured the effect of hookworms on otariid pup's growth (Chilvers et al., 2009; Delong, Orr, Jenkinson, & Lyons, 2009; Marcus et al., 2015). In addition to the resources extracted from the host by the parasite, there could be a significant amount of energy devoted to immune function (Sheldon & Verhulst, 1996). In otariids, hookworm infection elicits a strong immune response capable of clearing infection after 6–10 weeks of the initial infection (Seguel, Montalva et al., 2018). Therefore, it is possible that *Uncinaria* sp. induces “trade‐offs” in otariids between growth and the immune function, because the animals have to allocate more energy resources to clear the infection instead of growth (Budischak, O'Neal, Jolles, & Ezenwa, 2017). However, despite this decrease in growth rate, hookworm‐infected animals did not have a significantly different SMI compared to noninfected pups. Since the SMI is a measure of the proportion between length and weight, the lack of changes in this index but reduced growth rate suggest that pups with severe hookworm infection stop growing but do not lose a significant amount of body mass. In multimodel analyses, SMI was a more significant predictor of the level of water activity than growth rate, suggesting that, as shown in other studies, the beginning of water behavior is associated with body size in otariid pups (McCafferty et al., 1998; Pitcher et al., 2005; Richmond, Burns, & Rea, 2006).

The low hemoglobin concentrations in hookworm‐infected pups are explained because *Uncinaria* sp. is a hematophagous (i.e., blood‐sucking) nematode, and the anemic condition of infected pups is an indirect indicator of the extraction of resources from the host (Seguel, Muñoz et al., 2018). Additionally, hemoglobin is a key element for oxygen transport and a limiting factor in the development of swimming skills in pinnipeds (Kooyman & Ponganis, 1998). Thus, those pups with a high hookworm burden will have low hemoglobin concentrations and, therefore, lower transport capacity of oxygen in blood. Furthermore, since hemoglobin levels increase with the animal scaled body mass, it makes resource allocation more efficient for learning how to swim as the animals grow (Downs et al., 2013; Gastebois et al., 2011; Jeglinski, Werner, Robinson, Costa, & Trillmich, 2012; Spence‐Bailey, Verrier, & Arnould, 2007). Along the same line, and similar to other studies on pinniped swimming ontogeny, the most important predictors of the level of active water behavior were hemoglobin levels and SMI (Pitcher et al., 2005; Richmond et al., 2006). However, in the same analyses, hookworm burden did not have a significant effect in the presence of swimming events, and when the proportion of time spend in land versus water was analyzed, pups with severe hookworm infection were more likely to be observed in the water. Additionally, a higher proportion of hookworm‐infected pups engaged in swimming compared to noninfected pups. These observations are the opposite of our predicted impact of hookworms in the development of swimming skills given the known effects of hookworms on body mass and hemoglobin levels (Seguel & Gottdenker, 2017). An alternative explanation for these results can be given by a potential compensatory mechanism deployed by fur seal pups with hookworm infection. Since a significant number of pups suffer the adverse consequences of parasitism in terms of growth and anemia, they could compensate by spending more time close to water and engage in swimming, in order to develop skills that will be physiologically more challenging for these animals compared to nonparasitized animals. However, this compensatory behavior would demand a complex and strong selective pressure. There is little evidence in mammals that such mechanisms have been favored, although some animals can exhibit abnormal behavior in response to parasitism that is considered adaptive in terms of the host fitness such as decrease in manifestation of stress and pain (Brown, 1999; Poulin, 1994; Thomas, Poulin, & Brodeur, 2010). In other circumstances, these behavioral changes are associated with an advantage for the parasite in terms of transmission (Vanderwaal & Ezenwa, 2016). In the case of fur seals, an increase in time spent in water could be a compensatory mechanism to increase the likelihood of survival in the pups. Since hookworm transmission in otariids depends on the reproductive success of the host due to exclusive lactogenic transmission (Lyons et al., 2011; Seguel & Gottdenker, 2017), enhanced water activity could result in a transmission advantage for the parasite if this behavioral trait is related to better survival of the host, as shown in several otariid species (Blanchet, Lydersen, Ims, & Kovacs, 2016; Verrier et al., 2011). Hookworms could also impose selective pressure in otariids through increased survival of heterozygous animals to acute infection (Acevedo‐Whitehouse et al., 2006), which could have increased recruitment and better reproductive output (Forcada & Hoffman, 2014), increasing the chances of transmission for the parasite. These processes could have long‐term effects in the population and along with the present study highlight that the effects of parasitism are more complex at the population versus individual level.

Hookworm infection also alters the social behavior of pups, since hookworm‐infected pups on land decreased their level of social activity and they were less likely to be observed playing in water, a behavior associated with the formation of “spots” or social groups. Increases or decreases in social behavior due to parasitism are well documented in several taxa, but the mechanisms that drive these changes are not well understood (Ezenwa et al., 2016; Herbison, Lagrue, & Poulin, 2018). In this system, we predicted that social behavior could be decreased due to the detrimental effects of hookworms in energy balance and hemoglobin levels, which could restrain the movement of sick pups. However, whether a decrease in social behavior alters swimming ontogeny in pinnipeds is not clear, particularly if animals that decrease social behaviors engage in more active swimming behaviors as observed in this study.

Another potential explanation for the contrasting effects of hookworms on water behavior of fur seal pups is that the number of events in water or the type of water activity is the reflective of other traits related with hookworm infection, such as maternal care patterns. In most studied pinniped species, maternal care patterns strongly influence growth and body condition as well as swimming ontogeny by modifying the pup's activity budget (Arnold & Trillmich, 1985; Chilvers, Wilson, & Hickling, 1995; Doidge & Croxall, 1989; McDonald, Goebel, Crocker, & Costa, 2012; Jeanniard‐du‐Dot et al., 2017). Similarly, in our study, pups predominately engaged in water activities when females were not in the rookery, and the multimodel analysis revealed that the number of maternal care events negatively impacted the number of water events recorded in a pup. This suggests that pups receiving more maternal care spend less time in the water during their early development. SAFS pups that receive less maternal care have a less reactive immune system, clear hookworm infection later and tend to have higher hookworm burdens (Seguel, Montalva et al., 2018). This could explain why pups with higher burdens are more likely to be observed in water, because they spend less time with their mothers. The fact that pups treated with anti‐parasitic drugs showed intermediate levels of time investment in water could be due to the type of selection of this group. Since animals were randomly selected, it is very likely that there were animals in this group that could have developed low and high parasitic burdens as determined by their maternal care patterns. Therefore, this group likely contained animals with high and low levels of maternal attendance which influenced the time available for pups to develop their aquatic behaviors.

The presented evidence points toward an indirect connection between parasitism and aquatic behavior. However, regardless of whether there is an actual direct manipulation of host behavior by the parasite or a connection through the patterns of maternal care, the final results show that despite suffering severe decreases in growth rates and anemia, pups with higher burdens are able to compensate the theoretical physiological handicap for water activity due to low hemoglobin and growth rate by spending more time near water and engaging more often in swimming compared to noninfected pups. As shown in the multimodel analyses, the effect of parasite burden or ivermectin treatment was not significant in the overall level of activity in water or swimming, regardless of the known negative effects of hookworms on physiological traits important for the development of water behavior in otariids.

This work shows the contrasting effects of parasitism on aquatic animal populations and presents an interesting phenomenon of natural mitigation on the detrimental effects of parasites on the host. This study also furthers our understanding on how diseases in aquatic organisms have consequences beyond direct mortality.

## AUTHORS' CONTRIBUTIONS

F.M and M.S designed the study; F.M., D. P., J.G., and M.S. performed the field and laboratory work; F.M and M.S wrote the paper. All authors contributed critically to the drafts and gave final approval for publication.

## Data Availability

Raw data that originated the presented results will be archived as xlsx document in Dryad (10.5061/dryad.mc24m6j)
