# Supplementary material for: The contrasting hidden consequences of parasitism: Effects of a hematophagous nematode (Uncinaria sp.) in the development of a marine mammal swimming behavior
Source: Ecol Evol. 2019 Mar 7;9(7):3689–99. doi: 10.1002/ece3.4914 (PMC6468065; doi:10.1002/ece3.4914)
Supplement: Supplementary file 1 [file ECE3-9-3689-s001.docx]

Supplementary Table 1. Dive times, scaled body mass (SBM) and hemoglobin levels for *A. australis* pups.

| ID | Dive time within 10 minutes (min) | SBM | Hg (g/dL) |
| --- | --- | --- | --- |
| 90 | 1,54 | 14,8 | 12,3 |
| 14 | 2,14 | 12,7 | 7,8 |
| 1 | 3,04 | 11,1 | 9,5 |
| 108 | 3,17 | 12,5 | 11,2 |
| 96 | 3,32 | 11 | 10,1 |
| 91 | 3,39 | 11,6 | 13,6 |
| 100 | 3,41 | 10,8 | 10,4 |
| 124 | 5,06 | 10,8 | 6,9 |
| 120 | 5,31 | 13,4 | 11,5 |
| 9 | 5,33 | - | - |
| 20 | 8,37 | - | - |
